# Ultrasound Attenuation Coefficient as a Biomarker of Hepatic Steatosis: State of the Art and Software Evaluation

**DOI:** 10.3390/jcm15051816

**Published:** 2026-02-27

**Authors:** Giorgio Esposto, Jacopo Iaccarino, Sara Camilli, Linda Galasso, Rosy Terranova, Manuela Pietramale, Raffaele Borriello, Irene Mignini, Maria Elena Ainora, Antonio Gasbarrini, Maria Assunta Zocco

**Affiliations:** CEMAD Centro Malattie dell’Apparato Digerente, Medicina Interna e Gastroenterologia, Dipartimento di Medicina e Chirurgia Traslazionale, Università Cattolica del Sacro Cuore, Fondazione Policlinico Universitario “A. Gemelli” IRCCS, 00168 Rome, Italy; giorgio.esposto@guest.policlinicogemelli.it (G.E.); jacopo.iaccarino01@icatt.it (J.I.); sara.camilli01@icatt.it (S.C.); linda.galasso@guest.policlinicogemelli.it (L.G.); rosy.terranova01@icatt.it (R.T.); manuela.pietramale01@icatt.it (M.P.); raffaeleborr@gmail.com (R.B.); irene.mignini@guest.policlinicogemelli.it (I.M.); mariaelena.ainora@policlinicogemelli.it (M.E.A.); antonio.gasbarrini@unicatt.it (A.G.)

**Keywords:** attenuation coefficient, quantitative ultrasound, abdominal ultrasound, tissue characterization, ultrasound biomarkers

## Abstract

**Background/Objectives:** The attenuation coefficient (AC) is a quantitative ultrasound parameter that describes the frequency-dependent reduction of acoustic energy as ultrasound waves propagate through biological tissues. Recently, AC has gained increasing relevance in abdominal ultrasound as an objective and reproducible biomarker for tissue characterization, particularly in the assessment of diffuse parenchymal diseases. Unlike conventional qualitative B-mode imaging, AC provides standardized numerical measurements that improve interobserver reproducibility and facilitate longitudinal monitoring. **Methods:** This review provides a comprehensive and critical overview of the current clinical applications of AC measurements in abdominal ultrasound, mainly focusing on liver steatosis quantification. Emphasis is placed on the comparative evaluation of commercially available AC-based technologies, highlighting their methodological differences, validation evidence, and diagnostic performance to support future efforts toward harmonization and standardization across ultrasound platforms. **Results:** Several studies have demonstrated a strong correlation between AC values and established reference standards, including magnetic resonance imaging–proton density fat fraction (MRI-PDFF) and histopathological grading, supporting its role in the noninvasive evaluation of liver steatosis. The growing clinical adoption of AC has been accompanied by the development of multiple vendor-specific software implementations integrated into modern ultrasound systems. Although these platforms share a common physical basis, they differ substantially in algorithmic design, signal processing strategies and region-of-interest selection. These differences may influence absolute AC values and diagnostic cutoff thresholds, therefore limiting direct comparability across systems. Another factor that further contributes to the heterogeneity of reported cutoff values is the variability in validation approaches, with some technologies validated against liver biopsy and others against MRI-PDFF. **Conclusions:** AC is a promising quantitative ultrasound biomarker for noninvasive liver steatosis assessment, showing strong correlation with histology and MRI-PDFF. However, inter-vendor variability currently limits cross-platform comparability. Standardized acquisition protocols, unified quality-control criteria, phantom-based cross-calibration, and consistent vendor-specific reporting are essential to ensure reliable longitudinal monitoring and broader clinical implementation.

## 1. Introduction

Ultrasound imaging represents one of the most widely used and versatile diagnostic modalities in modern medicine, characterized by its real-time imaging capability, portability, cost-effectiveness, and lack of ionizing radiation [1]. Despite these advantages, conventional B-mode ultrasound remains fundamentally qualitative, relying on subjective visual interpretation of grayscale images with limited sensitivity, for example, for mild hepatic steatosis (below 50%) [2]. These limitations have driven the development of quantitative ultrasound (QUS) techniques, like the attenuation coefficient (AC) [3,4], which aim to provide objective and reproducible numerical biomarkers for tissue characterization.

AC describes the frequency-dependent loss of acoustic energy as ultrasound waves propagate through biological tissues and it is expressed in dB/cm/MHz [5]; it holds significant diagnostic value across a range of clinical applications, including tumor differentiation and the assessment of hepatic fat content [6].

Recent guidelines from major professional societies have recognized the clinical utility of attenuation-based quantification. The 2025 American Association for the Study of Liver Diseases (AASLD) guidelines acknowledge that steatosis quantification incorporated into conventional ultrasound machines is becoming more widely available, though noting that MRI-PDFF maintains superior diagnostic accuracy [7]. The World Federation for Ultrasound in Medicine and Biology (WFUMB) 2021 position paper on liver fat quantification highlighted attenuation coefficient estimation as one of the most clinically validated quantitative ultrasound (QUS) techniques, alongside controlled attenuation parameter (CAP) [8]. Notably, AC provides a fat-specific biomarker that is independent of other hepatic pathological processes. However, while AC is commonly described as fibrosis-independent in early and moderate stages, caution may be warranted in patients with advanced fibrosis or cirrhosis, where acoustic property alterations could influence attenuation measurements [9,10].

This review provides a comprehensive and critical analysis of the current state of AC in abdominal ultrasound, with particular emphasis on hepatic steatosis and comparative evaluation of commercial software platforms. We examine the technical and physical principles underlying AC measurement, review validated clinical applications with a focus on hepatic steatosis assessment and analyze differences in algorithmic implementation and performance across available platforms.

## 2. Methods

A search of the PubMed/MEDLINE database was performed up to December 2025. The search strategy included the following keywords and their combinations: “attenuation”, “attenuation coefficient”, “quantitative ultrasound”, “multiparametric liver ultrasound”, “AC” and “CAP”, together with “MASLD”, “hepatic steatosis”, “liver steatosis” and “abdominal imaging.” Reference of relevant articles were reviewed to identify additional studies. Only publications in English were considered. Three independent reviewers assessed eligibility. Only studies evaluating the use of attenuation-based technologies in abdominal imaging, with a particular focus on the assessment of hepatic steatosis, were included. A total of 82 articles were ultimately included in this review.

## 3. Technical and Physical Principles of the Attenuation Coefficient

As ultrasound propagates through biological tissue, the energy carried by the acoustic beam progressively decreases. This phenomenon is quantified by AC, which describes the rate at which the amplitude or intensity of an ultrasound wave diminishes with propagation distance [11,12].

Attenuation arises from two main mechanisms: absorption, whereby the mechanical energy of the wave is converted into heat, and scattering, which redistributes energy away from the primary direction of propagation. Together, these processes lead to a gradual reduction in signal strength as depth increases. The AC is typically expressed in decibels per centimeter per megahertz (dB/cm/MHz) and varies across tissues, reflecting differences in composition and microstructure [11]. For this reason, attenuation measurements provide valuable insight into tissue properties.

Accurate interpretation of AC, however, requires careful consideration of several influencing factors. Transducer frequency is among the most critical. In soft tissues, attenuation increases approximately linearly with frequency: higher frequencies undergo greater energy loss per unit distance due to enhanced absorption and scattering [13]. As a result, frequency selection directly affects AC estimation, as higher frequencies improve spatial resolution but reduce penetration depth. For example, liver tissue exhibits an average attenuation of about 0.45 dB/cm/MHz [14].

The depth at which measurements are performed also plays a significant role. As depth increases, estimated AC values tend to decrease, and the maximum usable depth is often limited by the signal-to-noise ratio. Several studies have addressed this issue by applying filtering techniques to suppress background noise [15]. This depth dependence underscores the importance of standardized acquisition protocols, including fixed regions of interest (ROIs) and predefined measurement depths [16,17].

In addition, the orientation of the ultrasound beam relative to anatomical structures must be carefully controlled. Variations in the angle of incidence affect reflection, refraction, and scattering, altering the amount of energy transmitted and received by the transducer and ultimately influencing AC estimates. Precise probe alignment is therefore essential for accurate and reproducible measurements [18].

Current efforts toward the standardization and clinical validation of AC in abdominal ultrasound focus on several key methodological aspects.

In recent years, numerous phantoms based on tissue-mimicking materials (TMMs) have been developed to closely replicate the acoustic properties of human tissues. These phantoms are fundamental for system calibration, ultrasound quality control, and the metrological validation of AC measurement techniques. To be effective, they must exhibit reproducible acoustic properties, long-term stability, and consistent behavior across defined frequency and temperature ranges [19,20]. A wide range of materials is used for phantom construction, including gelatin, agar, condensed milk-based gels, polyvinyl alcohol (PVA), and silicone, each offering acoustic properties suited to specific tissue types. Among these, agar-based phantoms are particularly advantageous because they display an approximately linear relationship between attenuation and frequency, closely mirroring the behavior of biological tissues and making them especially suitable for system calibration [20].

Another major source of variability in AC estimation arises from the diversity of available computational methodologies. Spectral difference methods estimate attenuation by comparing signal weakening with depth through analysis of frequency spectra acquired from two ROIs positioned at different depths [21]. Spectral shift methods, in contrast, exploit the progressive downward shift of the spectral centroid with depth, as higher frequencies are attenuated more rapidly than lower ones [5]. The spectral log-difference approach computes differences between the logarithms of power spectra measured at two axial locations and derives AC from the slope of a linear regression across frequencies [22,23,24]. Hybrid techniques combine spectral difference methods with normalization to a reference phantom, followed by spectral shift analysis to estimate frequency displacement with depth [25]. Comparative studies have shown that spectral difference methods provide the highest accuracy and precision in homogeneous ROIs but perform poorly when scatterer density varies. In contrast, log-difference and hybrid approaches remain robust under varying scatterer densities, although all methods show reduced reliability when scatterer size changes [26]. More recent algorithms incorporating automatic segmentation further improve performance by excluding atypically strong echoes, thereby reducing bias in heterogeneous tissues. These approaches have demonstrated accuracy improvements of up to 80–90% in inhomogeneous phantoms and good robustness in vivo, with reductions in mean AC estimates of approximately 5–25% and decreases in standard deviation of 30–50% across different measurement locations [27].

To further address variability related to ROI placement, confidence maps have been introduced to identify regions with optimal signal quality and to suggest ideal measurement depths. In liver applications, for example, AC is commonly measured within an ROI positioned approximately 2.5–4 cm below the liver capsule, in reference to AIUM–RSNA-QIBA Pulse-Echo Quantitative Ultrasound Biomarker Committee [28,29,30]. The use of acoustic phantoms with stable and well-defined properties remains crucial for routine system calibration and for ensuring comparability across different platforms [12,29]. Future studies on liver-mimicking phantoms, representative of the target condition (i.e., MASLD) are needed to establish the optimal depth for AC measurement. Although inter-system variability persists, the implementation of consistent protocols results in excellent intra-system and inter-operator reproducibility [31,32].

## 4. Comparative Analysis of Existing Software Tools

As highlighted above, variability among commercially available software and across different studies may limit the identification of universal cutoff values for diagnosis and longitudinal follow-up. Table 1 summarizes the cutoff values identified by each manufacturer.

The most extensively analyzed and validated technology to date is CAP, which was integrated into the liver fibrosis quantification technology of the FibroScan system (Echosens, France) in 2010. CAP quantifies hepatic fat content by measuring the attenuation of the amplitude of ultrasonic waves as they pass through the liver, providing values expressed in dB/m within a range of 100–400 dB/m [33,34,35,36]. The cutoff values identified by large multicenter European studies are applicable both to the M and XL probes [35]. CAP benefits from the largest validation database currently available, including individual patient data meta-analysis and extensive biopsy-correlated studies across diverse liver disease etiologies, patients’ BMI and steatosis severity [37,38]. This robust evidence base has led to widely adopted histology-based cut-offs and facilitated inclusion in international guidelines. The main limit of CAP is that it requires a dedicated probe, contrary to ultrasound attenuation technologies [39].

Compared to CAP, most of the other technologies express the AC in decibels per centimeter per megahertz (dB/cm/MHz), as this unit reflects the physical dependence of ultrasonic attenuation on both the propagation distance of the wave (cm) and its frequency (MHz). These Attenuation-based ultrasound techniques, which include Attenuation Imaging (ATI) by Canon Medical, UGAP by GE Healthcare, iATT/ATT by Fujifilm-Hitachi, and Ultrasound Steatosis Attenuation Technology (USAT) by Mindray have demonstrated strong correlations with histological steatosis grading and MRI-PDFF, with biopsy-validated studies commonly reporting threshold values of approximately 0.63–0.66 dB/cm/MHz for the detection of ≥S1 steatosis, 0.72–0.74 dB/cm/MHz for ≥S2, and ≥0.80–0.82 dB/cm/MHz for S3 steatosis [37,40,41,42,43,44,45,46,47,48,49].

Philips Liver Fat Quantification (LFQ) is based on a similar attenuation-driven methodology, with preliminary investigations indicating comparable diagnostic performance and cut-off values when referenced to MRI-PDFF [28], despite the monocentric nature of the study and the low sample size. By contrast, Ultrasound-Derived Fat Fraction (UDFF) by Siemens Healthineers applies a multiparametric algorithm that combines attenuation and backscatter information to directly estimate liver fat content as a percentage, just like MRI-PDFF, showing indeed a good agreement between the two techniques, with proposed thresholds of approximately 10–12% for mild, 15–20% for moderate, and greater than 27% for severe steatosis [50]. Samsung Medison adopts a hybrid strategy which combines both Tissue Attenuation Imaging (TAI) and ultrasound-derived fat fraction (USFF); however, standardized histology-based cut-offs for these parameters remain insufficiently established in large, biopsy-controlled cohorts [51,52,53,54,55]. An additional attenuation-based approach, different from the other mentioned above, is that introduced by Esaote (Q-Attenuation Imaging—QAI). It generates a real-time attenuation map over the liver parenchyma, allowing the operator to visually assess spatial homogeneity and place measurements within a color-coded attenuation field, potentially improving operator confidence in selecting representative parenchyma [56]. To date, QAI has not undergone robust peer-reviewed validation, and current evidence is limited to data presented at European ultrasound conferences. SuperSonic Imagine systems integrate both attenuation-based mapping (Att.PLUS) and sound-of-speed measurement (SSp PLUS), the latter reflecting variations in ultrasound propagation velocity associated with increased hepatic fat content; despite encouraging preliminary data, sound-of-speed-based steatosis quantification still lacks large-scale biopsy- or MRI-PDFF–validated cut-off definitions [57,58,59].

**Table 1:** Comparison of available technologies for Attenuation Coefficient. ATI, iATT, ATT, and UGAP denote vendor-specific implementations of AC.

Manufacturer	Technology	Unit of Measurement	Reference Cutoffs	Reference Standard
Echosens [35]	Controlled Attenuation Parameter (CAP)	dB/m	S1 ≥ 248 S2 ≥ 268 S3 ≥ 280	Histopathology
GE Health Care [45]	Ultrasound-Guided Attenuation Parameter (UGAP)	dB/cm/MHz	S1 ≥ 0.65 S2 ≥ 0.71 S3 ≥ 0.77	MRI-PDFF
Canon Medical [57]	Attenuation Imaging (ATI)	dB/cm/MHz	S1 ≥ 0.64 S2 ≥ 0.72 S3 ≥ 0.75	MRI-PDFF
Esaote [56]	Q-Attenuation Imaging (QAI)	dB/cm/MHz	S1 ≥ 0.60 S2 ≥ 0.70 S3 ≥ 0.77	Histopathology
Mindray [47]	Ultrasound attenuation analysis technique (USAT)	dB/cm/MHz	S1 ≥ 0.53 S2 ≥ 0.62 S3 ≥ 0.82	Histopathology
SuperSonic Imagine [60,61]	Att.PLUS SSp PLUS	dB/cm/MHz m/s	S0–S1: Att < 0.45 and SSp > 1524 S2–S3: Att > 0.45 and SSp < 1524	MRI-PDFF
Philips [28]	Liver Fat Quantification (LFQ)	dB/cm/MHz	S1 ≥ 0.61 S2 ≥ 0.68 S3 ≥ 0.74	MRI-PDFF
Samsung Medison [55]	TAI USFF	dB/cm/MHz %	S1 ≥ 0.65 S2 ≥ 0.77 S3 ≥ 0.79	CAP
Siemens Healthineers [50]	Ultrasound-Derived Fat Fraction (UDFF)	%	S1 ≥ 10–12% S2 ≥ 15–20% S3 ≥ 27%	MRI-PDFF
Fujifilm Hitachi [62]	iATT	dB/cm/MHz	S1 ≥ 0.74 S2 ≥ 0.79 S3 ≥ 0.81	MRI-PDFF

Variability in reported AC cut-off values among US systems primarily reflects technical and methodological differences rather than true discrepancies in liver fat content (Table 2). Although all AC-based methods are based on the same physical principle of frequency-dependent acoustic attenuation, each manufacturer has its own algorithm, deploys different spectral analysis techniques, calibration strategies, and quality assurance criteria. Moreover, differences in ROI placement, depth selection, ROI size, and artifact exclusion may further contribute to systematic inter-platform variation. The selected reference standard also impacts threshold determination. Indeed, cut-offs derived against MRI-PDFF tend to be more consistent and generalizable across populations compared to biopsy-based thresholds, which can vary depending on sampling location and interobserver variability across pathologists.

To address variability in AC cut-off values, several strategic measures should be implemented. First, vendor-specific reference values should be established and clearly reported, recognizing that AC measurements are not interchangeable across platforms. Standardized acquisition protocols—including uniform ROI placement, depth selection, and probe settings—are essential to improve comparability. Likewise, harmonized quality control criteria should be adopted to enhance measurement reproducibility. For longitudinal assessment, follow-up examinations should be performed using the same ultrasound system and probe to avoid artificial variability. In biopsy-referenced studies, efforts should focus on minimizing sampling error and interobserver variability through standardized histologic evaluation. Greater reliance on MRI-PDFF as a reference standard may further improve reproducibility across technologies. Large multicenter validation studies are necessary before clinical implementation. Finally, international consensus guidelines, cross-calibration strategies, and technical standardization initiatives are needed to reduce inter-platform discrepancies and to define more consistent diagnostic thresholds for clinical practice.

## 5. Current Clinical Utility of the Attenuation Coefficient

### 5.1. Validated Applications for Steatosis’ Assessment

**Figure 1:**
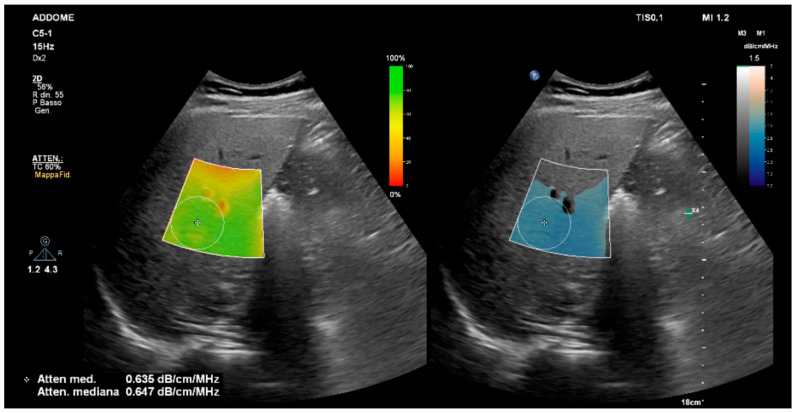
AC values suggestive of liver steatosis grade S1—Philips LFQ. AC: attenuation coefficient; LFQ: liver fat quantification; S1: stage 1.

The AC is currently one of the most mature and clinically validated quantitative ultrasound parameters for abdominal imaging and represents the cornerstone of contemporary ultrasound-based assessment of hepatic steatosis. Compared with conventional qualitative B-mode ultrasound, AC provides objective and quantitative metrics that significantly improve the detection and grading of hepatic steatosis, particularly in the setting of mild to moderate disease, which remains challenging for visual assessment alone (Figure 1) [63]. A large set of studies has validated AC against MRI-proton density fat fraction (MRI-PDFF), which is considered the non-invasive gold standard for liver fat quantification. Attenuation coefficient measurements demonstrated strong linear relationships with MRI-PDFF and consistently high diagnostic accuracy for both detection and grading of hepatic steatosis, including mild disease [39]. Tanpowpong et al. extended this evidence by providing MRI-PDFF-referenced cut-off values for the Att.PLUS technique in 162 patients with NAFLD, identifying thresholds of 0.46, 0.50, and 0.52 dB/cm/MHz for ≥S1, ≥S2, and ≥S3 steatosis, respectively, with AUROC values ranging from 0.70 to 0.82 [64]. These findings have been reinforced by subsequent prospective studies performed across different ultrasound vendors and technical implementations, demonstrating robust diagnostic performance [62]. Cassinotto et al. underscored the robustness and generalizability of AC by comparing three different ultrasound systems and showing that AC-based metrics achieved high diagnostic performance for MRI-PDFF–defined steatosis with AUROC values of approximately 0.88–0.94 for ≥S1 and ≥S2 supporting the generalizability of AC across platforms [61]. Although most studies have reported excellent diagnostic performance, substantial variability in proposed cut-off values has been observed across studies. This variability may reflect differences in measurement protocols and manufacturer-specific algorithms, as well as heterogeneity in study populations, inclusion criteria, and disease prevalence [3]. Moreover, AC has also consistently shown comparable or superior diagnostic accuracy compared with controlled attenuation parameter (CAP) when MRI-PDFF is used as the reference, particularly for mild steatosis and for steatosis grading [57,58,59]. Additional head-to-head analyses against other ultrasound-derived quantitative parameters have confirmed that AC ranks among the best-performing biomarkers particularly in the detection of mild steatosis [57,65,66]. However, the robustness of AC as a biomarker is further supported by multiple biopsy-proven cohorts. Early work from Koizumi et al. in 94 patients with chronic liver disease who underwent both liver biopsy and quantitative attenuation measurement (ATT) on B-mode ultrasound showed that ATT increases stepwise with histological steatosis grade and provides good diagnostic accuracy for ≥S1 and ≥S2 steatosis, while also performing at least comparably to the controlled attenuation parameter (CAP) obtained with vibration-controlled transient elastography [58]. Subsequent biopsy-proven studies confirmed the technical feasibility of AC in routine clinical settings and consistently reported strong correlations between AC values and histologic fat content, with reliable discrimination between different steatosis severity categories across Metabolic dysfunction-associated steatotic liver disease (MASLD) populations [10,59]. Li et al. addressed a key methodological issue by investigating the optimal number of valid AC measurements in 139 biopsy-proven MASLD patients. Their results highlighted that diagnostic performance plateaued after a limited number of high-quality acquisitions, indicating that streamlined acquisition protocols can preserve accuracy while reducing examination time and operator dependency [67]. A novel study compared AC-Canon (ATI) and CAP in patients with type 2 diabetes and MASLD, using MRI–PDFF and histology as reference standards. ATI outperformed CAP for detecting steatosis (S > 0) when MRI–PDFF was used, while no differences were observed with histology as the reference [68]. More recently, Hobeika et al. pooled individual patient data from multiple biopsy-proven cohorts to derive and externally validate a 2D AC-based steatosis grading system, confirming that AC retains high discriminative ability across different populations and imaging setups [67]. A meta-analysis of 13 studies comprising 1509 patients assessed attenuation coefficient (AC) algorithms from multiple manufacturers. Pooled sensitivity and specificity were 76% and 84% for detecting steatosis ≥S1 (histology or PDFF) and 87% and 79% for detecting steatosis ≥S2, with hierarchical summary AUCs of 0.83 and 0.91, respectively [69]. To date, only three technologies have been validated against MRI-PDFF on large multicenter studies: UGAP by GE [45], ATI by Canon [57] and iATT by Fujifilm [62]. Hirooka et al. evaluated the iATT technique in a large cohort of patients from seven centers who underwent on the same-day ultrasound and MRI-PDFF. iATT values increased stepwise with MRI-defined steatosis grade, and ROC analysis demonstrated high diagnostic accuracy for steatosis detection and grading, with AUROC values consistently above 0.80 for ≥S1 and ≥S2 steatosis [62]. These results were corroborated by Nishimura et al. in a large prospective multicenter study conducted in 10 different centers, which demonstrated that AC was superior to CAP for both detection and grading of steatosis, with more consistent performance across centers [57]. Imajo et al. evaluated the performance of UGAP for grading hepatic steatosis against MRI-PDFF in a large multicenter study involving 1010 patients from seven different centers. Diagnostic performance was high across each steatosis grade with an AUROC for detecting any steatosis (MRI-PDFF ≥5.2%) of 0.910, for moderate steatosis (≥11.3%) of 0.912, and for severe steatosis (≥17.1%) of 0.894. Optimal UGAP cut-off values were approximately 0.65 for ≥S1, 0.71 for ≥S2, and 0.77 for S3, with good sensitivity and specificity for each threshold [46]. Importantly, attenuation coefficient measurements have also been validated in pediatric populations, where good correlation with MRI-derived fat fraction and satisfactory diagnostic accuracy for pediatric MASLD have been demonstrated, supporting extension of these techniques to younger patients [70]. Table 3 summarizes the main studies on the AC in MASLD patients. In conclusion, in studies using MRI-PDFF as reference standard, the AC consistently demonstrated high diagnostic performance, with AUROC values generally above 0.80 in multicenter cohorts [46,59,66,71]. In biopsy-based studies [58,72], variability may partly reflect sampling limitations and smaller sample sizes. Cross-platform comparisons showed broadly comparable discriminative ability, although cut-off values were not interchangeable due to proprietary algorithm implementation and platform-specific acquisition settings [59,71]. Multicenter investigations improved generalizability but also introduced protocol heterogeneity, whereas single-center studies often reported internally derived thresholds requiring external validation [58,64,70,72]. Overall, differences across studies appear to be driven more by technical and methodological factors than by intrinsic limitations of the AC itself.

**Table 3:** Overview of Major Studies on MASLD Using AC and MRI-PDFF or Biopsy as Reference Standard.

Study	Study Design and Sample Size	AC Technology	Reference Standard	Cut-Offs/Metrics	Sources of Variability	Key Findings	Sponsorship
Tanpowpong et al. [64]	Prospective single-center, 162 NAFLD patients	Att.PLUS	MRI-PDFF, S1–S3 thresholds defined according to PDFF values	≥S1: 0.46, ≥S2: 0.50, ≥S3: 0.52 dB/cm/MHz; AUROC 0.70–0.82	- Cut-offs derived in NAFLD-only cohort (spectrum bias) - Att.PLUS-specific implementation (no cross-platform calibration)	Provided MRI-PDFF–referenced thresholds; reliable grading of mild–moderate steatosis	Not reported
Cassinotto et al. [61]	Prospective multicenter, 226 NAFLD patients	AC (3 US systems)	MRI-PDFF for steatosis grading (predefined PDFF thresholds for ≥S1 and ≥S2).	AUROC ~0.88–0.94 for ≥S1 and ≥S2	- Three-platform comparison (algorithm-driven inter-platform variability) - PDFF thresholds fixed, but platform-specific ROI/depth constraints	Demonstrated generalizability across platforms	Investigator-initiated study
Koizumi et al. [58]	Prospective single-center, 94 chronic liver disease	ATT	Histopathology with steatosis standard histological criteria	Stepwise increase with histological grade	- Biopsy reference (sampling variability vs. imaging ROI) - Early-generation ATT implementation (limited generalizability)	Good diagnostic accuracy for ≥S1 and ≥S2; comparable to CAP	Not reported
Li et al. [72]	Prospective single-center, 139 MASLD patients	AC	Histopathology with steatosis standard histological criteria.	N/A	- Protocol study: outcome depends on “valid measures” definition - Biopsy reference + single-center acquisition workflow	Optimal number of measurements identified; accuracy plateaued early	Not reported
Hobeika et al. [67]	Meta-analysis, Pooled biopsy-proven cohorts	2D-AC	Histopathology and MRI-PDFF (pooled multicenter cohorts with external validation)	N/A	- Pooled heterogeneous acquisition protocols - Mixed reference standards/cohorts (derivation vs. external validation)	Developed and validated AC-based grading system	Not reported
Hirooka et al. [62]	Prospective multicenter, 273 patients	iATT	MRI-PDFF (quantitative steatosis thresholds)	AUROC >0.80 for ≥S1 and ≥S2	- Center-related acquisition variability - iATT vendor-specific QC/ROI rules (implementation-dependent)	Confirmed diagnostic accuracy and stepwise increase with steatosis	Investigator-initiated study
Nishimura et al. [57]	Prospective multicenter, 271 patients	ATI	MRI-PDFF (quantitative steatosis thresholds)	N/A	- ATI vs. CAP comparison (different physical metrics, non-interchangeable) - Multicenter PDFF workflow differences (scanner/protocol heterogeneity)	ATI superior to CAP; consistent performance across centers	Not reported
Imajo et al. [45]	Prospective multicenter, 1016 patients	UGAP	MRI-PDFF (quantitative steatosis thresholds)	≥S1: 0.65, ≥S2: 0.71, ≥S3: 0.77; AUROC 0.894–0.912	- Large-cohort UGAP: device/software versioning effects possible - PDFF protocol variability across sites (MRI harmonization)	High diagnostic performance across all steatosis grades	Not reported
D’Hondt A et al. [70]	Prospective single-center, 64 pediatric patients	AC	MRI-PDFF	N/A	- Pediatric cohort (different body habitus/acoustic path) - Sedation/breath-hold compliance variability (acquisition feasibility)	Good correlation and diagnostic accuracy; feasible in children	Not reported

### 5.2. Emerging and Investigational Uses of AC in Abdominal Imaging

Beyond its established role in hepatic steatosis assessment, the AC is increasingly being explored in a growing number of abdominal applications, where emerging evidence suggests a meaningful potential. In liver imaging, recent quantitative ultrasound studies have applied attenuation-based metrics to the characterization of focal liver lesions. Rafati et al. demonstrated that local attenuation coefficient slope imaging improves lesion conspicuity and discrimination between benign and malignant solid liver nodules, showing higher contrast-to-noise ratio and good diagnostic accuracy compared with conventional B-mode ultrasound [73]. Preliminary investigations have assessed attenuation-related parameters in renal disease. More recently, advanced quantitative ultrasound approaches incorporating attenuation-related metrics have been applied in the evaluation of fibrotic changes in kidney transplant recipients, demonstrating feasibility and correlation with histologic fibrosis [74]. Gray et al. reported the first systematic measurements of sound speed and attenuation in human pancreatic tissue and pancreatic tumors, demonstrating that human-specific attenuation values substantially affect acoustic modeling and thermal dose prediction in focused ultrasound therapies. These data highlight the relevance of accurate attenuation quantification for treatment planning and safety, while diagnostic applications in pancreatic disease remain largely unexplored [75]. However, beyond hepatic steatosis, evidence supporting clinical applications of the attenuation coefficient is still limited, with most data derived from small cohorts and lacking multicenter validation or guideline endorsement. Accordingly, current international guidelines do not yet recommend attenuation-based metrics for routine abdominal applications outside hepatic steatosis, and their use should presently be considered investigational.

### 5.3. Challenges and Limitations of AC Measurements

Despite the established clinical utility of ultrasound-derived attenuation coefficient (AC) for liver fat quantification, several technical, patient-related, and system-dependent limitations must be considered, as they directly affect measurement accuracy and reproducibility in real-world practice. Firstly, although AC substantially reduces subjectivity compared with qualitative B-mode ultrasound, a residual degree of operator dependency remains unavoidable. Variability related to probe orientation, intercostal versus subcostal approach, breath-hold execution, acquisition depth, and ROI size and positioning continues to influence measurement outcomes, underscoring the necessity for standardized acquisition protocols and real-time quality indicators. This was clearly demonstrated by Ferraioli et al., who reported good inter-operator agreement when standardized criteria were applied, but highlighted persistent variability when acquisition conditions deviated from recommended protocols [32]. Furthermore, beyond operator effects, heterogeneity in algorithmic design, signal modeling, and reporting formats across vendors contributes to inter-platform variability and represents a major challenge for universal standardization and inter-system comparability. Jeon et al. evaluated the inter-platform reproducibility of ultrasound attenuation measurements in patients with nonalcoholic fatty liver disease and found only moderate agreement between different ultrasound vendors, despite acceptable repeatability within the same system [76]. These findings were further corroborated by a subsequent study from the same work group, which demonstrated limited inter-platform interchangeability of ultrasound-based fat fraction estimates, reinforcing concerns regarding absolute value comparability across devices [77]. Moreover, depth dependence represents a well-recognized technical limitation of attenuation coefficient measurements. The increasing variability and reduced agreement of attenuation values at greater acquisition depths, particularly in individuals with increased subcutaneous fat thickness represent the main limitations. These findings suggest that attenuation measurements should preferentially be performed within optimized depth ranges to preserve accuracy and reproducibility [16,78]. In contrast to acquisition-related factors, physiological conditions such as food or water intake appear to have a negligible impact on attenuation coefficient measurements. In a two-center prospective study, Ferraioli et al. demonstrated that neither water ingestion (500 mL) nor a standardized meal (~600 kcal) resulted in significant changes in AC values when assessed up to 45 min after water intake and 120 min after meal consumption [79]. Finally, liver tissue composition may represent a biological confounder. Although the attenuation coefficient (AC) has generally been considered independent of fibrosis severity [80], emerging evidence indicates that advanced fibrosis may influence AC measurements and potentially affect the accuracy of steatosis estimation, thus requiring cautious interpretation in this subgroup of patients [81]. In particular, Kumada et al. demonstrated that advanced fibrosis could lead to an overestimation of steatosis when using attenuation-based ultrasound techniques, even in the absence of histologically confirmed fat accumulation. This effect is likely attributable to fibrosis-related alterations in tissue scattering and absorption properties. These findings underscore the importance of carefully interpreting AC values in patients with advanced fibrotic disease and support the integration of attenuation metrics with complementary methods for fibrosis assessment [81].

## 6. Conclusions

The AC represents a valuable and promising quantitative ultrasound tool that improves the objectivity and reproducibility of abdominal imaging, especially for noninvasive liver steatosis assessment. Its potential role in routine clinical practice is supported by strong evidence of correlations with histopathology and MRI-PDFF. Despite latest advancements, currently available AC-based technologies still differ in algorithmic implementation, ROI handling, and validation strategies. This variability accounts for the differences in absolute and cutoff values, limiting cross-vendor comparability. Although vendor-specific reference ranges remain necessary, future efforts should focus on standardized acquisition protocols, unified quality control criteria, and phantom-based cross-calibration strategies to improve inter-platform comparability. Consistent use of the same system for longitudinal follow-up is critical to ensure reliable monitoring, and routine clinical practice and reporting should explicitly include vendor-specific interpretative legends to ensure correct threshold application and avoid misclassification. Large multicenter studies with a broader adoption of MRI-PDFF as a reference standard may enhance reproducibility and support imaging-based alignment across technologies, while reducing limitations inherent to biopsy-based validation. Ultimately, international guideline development and coordinated standardization initiatives will be pivotal to facilitate consistent clinical implementation and more robust diagnostic integration of attenuation-based techniques.

## Figures and Tables

**Table 2:** Sources of variability affecting cut-off values in liver fat quantification.

Domain	Source of Variability	Mechanism	Impact on Cut-Off Values
Vendor Implementation	Proprietary algorithms	Spectral analysis, frequency modeling, slope estimation, internal calibration	Systematic shifts in AC across platforms
	ROI	Size, depth, vessel exclusion, spatial averaging	Measurement variability and platform-dependent scaling
	Quality control	Reliability metrics (stability index, IQR, signal strength filters)	Inclusion/exclusion of marginal measurements
	Hardware & transducer	Probe frequency, gain profiles	Frequency-dependent attenuation differences
Reference Standard	Biopsy	Histologic grading of small tissue samples	Cut-offs aligned to steatosis grades (e.g., ≥5%, ≥33%)
	MRI–PDFF	Whole-liver fat fraction measurement	Cut-offs aligned to volumetric fat percentage thresholds
Study Design Factors	Population characteristics	Differences in BMI, fibrosis stage, etiology	Population-specific threshold shifts
	Validation strategy	Biopsy-based vs. MRI-based calibration	Different definitions of steatosis grades

## Data Availability

No new data were created or analyzed in this study. Data sharing is not applicable to this article.
